# Classification of musculoskeletal pain using machine learning

**DOI:** 10.1038/s41598-025-12049-9

**Published:** 2025-07-25

**Authors:** Dalia Mohamed Fouad, Marwa Mahmoud Mahfouz, Mohammed Mostafa Mohamed, Mahmoud Yassin Elzanaty, Tarek Abd El-Hafeez

**Affiliations:** 1 Physical Therapy for Basic Science, Faculty of Physiotherapy, Deraya University, El-Minia, Egypt; 2 Physical Therapy for Biomechanics, Faculty of Physiotherapy, Sphinx University, Assiut, Egypt; 3 Physical Therapy for Neurology, Faculty of Physiotherapy, Deraya University, El-Minia, Egypt; 4 Cairo University, El-Giza, Egypt; 5 Computer Science Unit, Deraya University, El-Minia, Egypt; 6 Department of Computer Science, Faculty of Science, Minia University, El-Minia, Egypt

**Keywords:** Musculoskeletal pain, Particle swarm optimization (PSO), Neural networks, Classification, Personalized interventions, Clinical validation, Health care, Health occupations, Risk factors, Signs and symptoms, Computer science, Information technology, Scientific data

## Abstract

Musculoskeletal pain is a significant health concern affecting individuals across various demographics and professions, often leading to reduced productivity and impaired quality of life. This study proposes a framework leveraging Particle Swarm Optimization (PSO) to evaluate and assess musculoskeletal pain risk based on a comprehensive dataset encompassing demographic, professional, physical, and lifestyle characteristics. The dataset includes detailed information on individuals’ pain experiences across multiple body regions, providing a robust foundation for identifying correlations and risk factors. By integrating PSO with neural networks, this framework aims to enhance the detection of pain risk patterns, offering insights into the interplay between various factors and musculoskeletal health. The proposed framework involves data preprocessing, definition of neural network architecture, implementation of PSO, and performance evaluation. The dataset, containing 350 entries, was preprocessed to handle missing values, balance class distributions using SMOTE, and normalize features. A fully connected feedforward neural network with a single hidden layer was employed, with PSO optimizing the network’s weights and biases. Performance was evaluated using metrics including accuracy, precision, recall, F1-score, and AUC-ROC. The results demonstrate that the PSO-optimized neural network effectively identifies musculoskeletal pain risk, achieving strong performance across all evaluation metrics (accuracy 95.8–100%). Key determinants such as age, BMI, exercise frequency, and occupational factors were identified, providing valuable insights for targeted interventions. The framework’s performance compares favorably with conventional approaches, highlighting the potential of optimization techniques in musculoskeletal pain assessment and the development of preventive strategies.

## Introduction

Musculoskeletal disorders (MSDs) represent one of the most significant global health challenges, with low back pain (LBP) alone affecting approximately 619 million people worldwide in 2020, projected to rise to 843 million cases by 2050^1^. According to the 2020 Global Burden of Disease Study, LBP accounted for 69.0 million years lived with disability (YLDs), ranking as the leading cause of global disability. The overall burden of musculoskeletal conditions is even more staggering, affecting over 1.63 billion people and representing the second leading cause of non-fatal disabilities worldwide. These disorders encompass a wide spectrum of conditions, with prevalence rates varying significantly across populations. Among elderly individuals, musculoskeletal pain affects 65–85% of the population, with back pain specifically impacting 36–70%^1^. In working-age adults, 60–80% will experience LBP during their lifetime, with prevalence rates in the United States ranging from 10 to 30% at any given time and lifetime prevalence reaching 65–80%^2,3^. The economic consequences are equally profound. Musculoskeletal conditions create substantial healthcare burdens, with LBP alone ranking sixth in overall disease burden globally. These disorders particularly impact occupational populations, showing elevated prevalence among healthcare workers (75%), office employees (45%), and manual laborers (62%) according to recent epidemiological studies. Understanding these factors and their interactions is critical for developing effective prevention, management, and intervention strategies^4–6^.

The growing burden of musculoskeletal pain has prompted extensive research into its causes, risk factors, and potential solutions. Traditional approaches to studying musculoskeletal pain often rely on linear statistical models or conventional machine learning techniques, which may not fully capture the intricate, non-linear relationships between variables. For instance, while age, body mass index (BMI), and occupational factors are commonly associated with musculoskeletal pain, their interactions with lifestyle habits such as exercise frequency, work hours, and ergonomic practices are less understood. Moreover, existing studies frequently focus on isolated pain regions or specific populations, neglecting the holistic view necessary for comprehensive pain management. This limitation underscores the need for advanced analytical frameworks that can effectively model the complex interplay of factors contributing to musculoskeletal pain^7–9^.

**Fig. 1:**
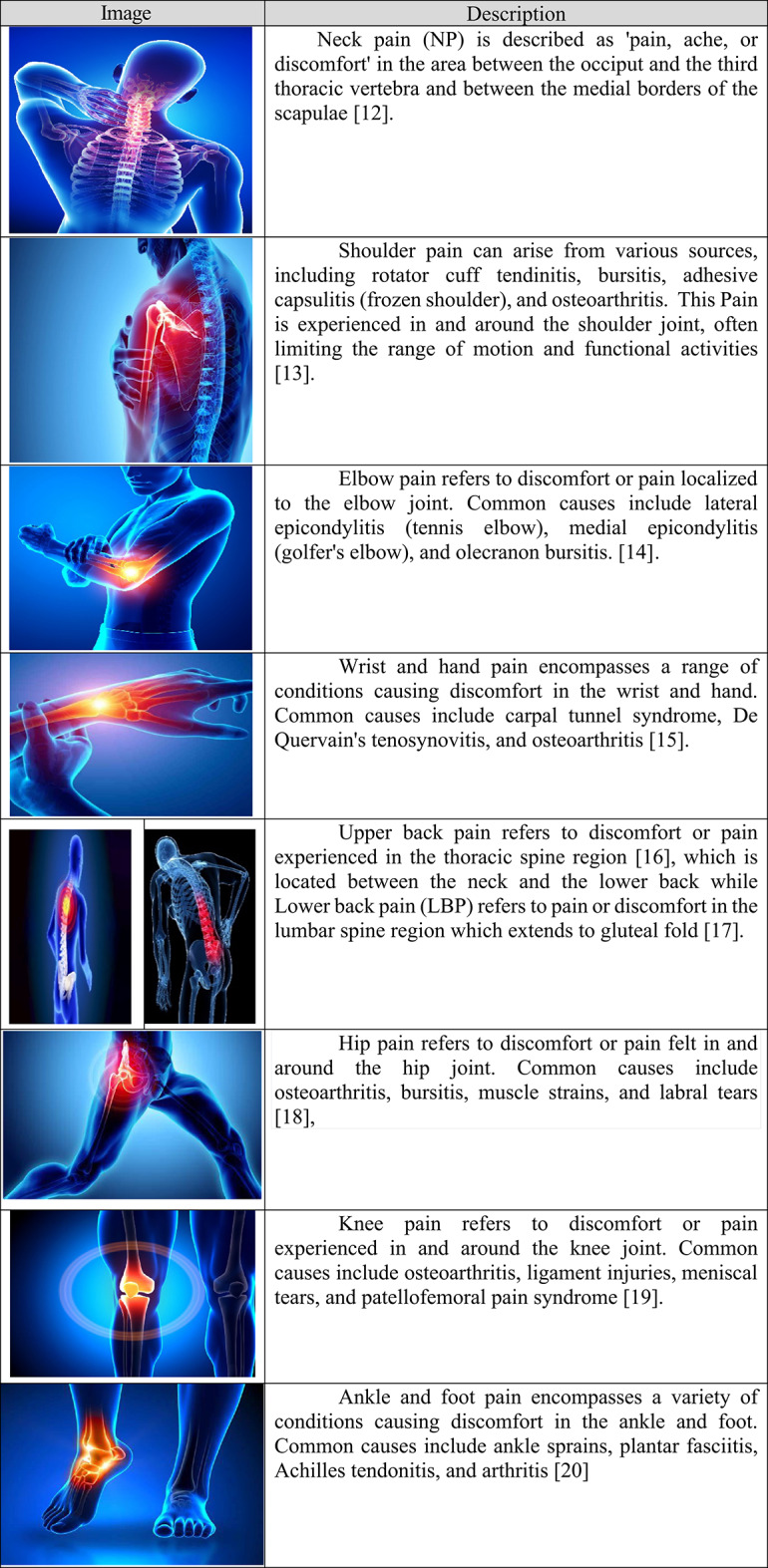
Body regions evaluated for musculoskeletal pain obtained from public website and no permission needed^21–23^.

Musculoskeletal disorders (MSDs) refer to conditions that affect the body’s support system, including muscles, bones, joints, tendons, ligaments, nerves, and surrounding connective tissues. These disorders can cause pain whether temporary or lifelong alongside reduced mobility and diminished dexterity, which ultimately restricts functional abilities and participation in daily life^10,11^. Figure 1 (obtained from public website and no permission needed^21–23^) .highlights the nine body areas assessed for work-related pain in the study. The evaluation included the neck, shoulders, upper back, elbows, wrists/hands, lower back, hips, knees, and ankles/feet - regions most commonly affected by musculoskeletal disorders in academic professionals. These areas were systematically examined to identify pain patterns and their potential links to teaching activities and work habits.

In recent years, machine learning and optimization algorithms have emerged as powerful tools for analyzing complex datasets and making accurate evaluation^24–26^. Among these, Particle Swarm Optimization (PSO)^27^ has gained prominence for its ability to efficiently explore large solution spaces and optimize complex functions. Inspired by the social behavior of bird flocking or fish schooling, PSO is a population-based stochastic optimization technique that balances exploration and exploitation to find optimal solutions. Its application spans various domains, including engineering, finance, and healthcare, where it has been used to optimize neural networks, feature selection, and diagnostic modeling. However, the use of PSO in musculoskeletal pain research remains underexplored, particularly in the context of integrating it with neural networks for pain evaluation. This study proposes a framework that leverages PSO to optimize neural network training for musculoskeletal pain classification. The framework is designed to address the limitations of traditional approaches by capturing the complex, non-linear relationships between demographic, professional, physical, and lifestyle factors. The dataset used in this study is comprehensive, encompassing information on individuals’ age, sex, professional rank, work hours, physical attributes (e.g., weight, height, BMI), lifestyle habits (e.g., exercise frequency, extra work), and pain experiences across multiple body regions. By integrating PSO with neural networks, the framework aims to enhance accuracy and provide deeper insights into the determinants of musculoskeletal pain.

The significance of this study lies in its potential to advance the understanding of musculoskeletal pain and improve diagnostic modeling in healthcare. By identifying key determinants of pain and optimizing classification models, the framework can support the development of targeted interventions and preventive strategies. For instance, healthcare professionals can use the insights gained from this study to design personalized exercise programs, ergonomic interventions, and workplace policies that reduce the risk of musculoskeletal pain. Policymakers can leverage the findings to promote public health initiatives aimed at improving musculoskeletal health across different populations. Additionally, individuals at risk of musculoskeletal pain can benefit from early detection and tailored recommendations based on their unique characteristics and lifestyle habits.

### Problem statement

Musculoskeletal pain is a multifaceted condition influenced by a combination of demographic, professional, physical, and lifestyle factors. Traditional approaches to analyzing and predicting pain often rely on linear models or conventional machine learning techniques, which may not fully capture the complex, non-linear relationships inherent in the data. Additionally, existing studies frequently focus on isolated factors or specific pain regions, neglecting the holistic view necessary for comprehensive pain management. There is a need for an advanced, optimized framework that can effectively analyze the intricate relationships between diverse variables and predict pain occurrences with high accuracy. This study addresses this gap by proposing a PSO-based framework for musculoskeletal pain classification, aiming to enhance the understanding of pain determinants and improve classification performance.

### Research question

How can Particle Swarm Optimization (PSO) be effectively integrated with neural networks to analyze and predict musculoskeletal pain based on a comprehensive dataset of demographic, professional, physical, and lifestyle characteristics?

### Research gap

While machine learning approaches for musculoskeletal pain assessment have been extensively studied, the application of advanced optimization techniques in this domain remains relatively underexplored. Gradient-based optimization methods are indeed the standard approach for neural network training, offering well-established advantages in convergence and computational efficiency. However, alternative optimization strategies like Particle Swarm Optimization (PSO) may offer complementary benefits worth investigating, particularly for specific problem configurations or when dealing with certain types of local optima. The current literature on musculoskeletal pain prediction has primarily focused on conventional machine learning architectures with standard optimization approaches. Few studies have systematically examined how hybrid approaches combining neural networks with bio-inspired optimization techniques might perform in this specific application domain. Our work explores this less-traveled path not as a replacement for gradient-based methods, but as a potential alternative worth evaluating in the context of pain prediction, where the nature of medical data and the importance of robust feature selection may present unique opportunities. Our contribution lies not in claiming a methodological gap in optimization techniques generally, but rather in investigating whether PSO-enhanced approaches might offer specific advantages for musculoskeletal pain prediction tasks. This is particularly relevant given the complex, multidimensional nature of pain-related data, where traditional approaches sometimes struggle to capture nonlinear relationships between diverse risk factors. The empirical results we present should be viewed as an exploration of this specific application rather than as a general challenge to established optimization practices.

### Contributions

1. Proposed framework: This study introduces a framework that integrates Particle Swarm Optimization (PSO) with neural networks for musculoskeletal pain classification. The framework is designed to optimize the training process, enhancing the model’s ability to capture complex relationships within the data.
2. Comprehensive dataset analysis: The study utilizes a detailed dataset that includes demographic, professional, physical, and lifestyle characteristics, providing a holistic view of factors influencing musculoskeletal pain. This comprehensive approach allows for a more accurate and nuanced analysis of pain determinants.
3. Optimized classification model: By employing PSO, the framework optimizes the weights and biases of the neural network, improving accuracy and robustness. This optimization process ensures that the model effectively balances exploration and exploitation, leading to strong performance.
4. Identification of key pain determinants: The framework identifies significant correlations and risk factors for musculoskeletal pain across various body regions, offering valuable insights for targeted interventions and preventive measures.
5. Performance evaluation: The study conducts a thorough evaluation of the proposed framework using multiple performance metrics, including accuracy, precision, recall, F1-score, and AUC-ROC. This comprehensive assessment demonstrates the framework’s effectiveness in predicting musculoskeletal pain.
6. Practical implications: The findings of this study have practical implications for healthcare professionals, policymakers, and individuals at risk of musculoskeletal pain. By identifying key determinants and optimizing classification models, the framework supports the development of tailored interventions and preventive strategies, ultimately improving musculoskeletal health outcomes.

## Related work

The application of artificial intelligence (AI) and machine learning (ML) techniques to predict, detect, and classify musculoskeletal disorders (MSDs) and low back pain (LBP) has become an active area of research. Various studies have explored different models, sensor types, and datasets to achieve progress in posture classification, pain assessment, and risk identification.

Several studies have concentrated on analyzing posture and movement using wearable sensor technology. For instance, Zemp et al.^28^ employed force and acceleration sensors to collect sitting posture data, reporting that a Random Forest algorithm achieved a mean accuracy of 90.9%. Conforti et al.^29^ utilized wearable sensors for biomechanical data collection during lifting tasks, with a Support Vector Machine (SVM) reportedly achieving 99.4% accuracy in distinguishing correct from incorrect lifting postures. Donisi et al.^30^ also used wearable inertial sensors for lifting task analysis, where tree-based algorithms reached accuracies exceeding 90% in binary risk classification. More recently, Rao^30^ developed an active orthosis for individuals with impaired trunk control using EMG and IMU data, achieving classification accuracies between 87.0% and 95.44%. While these studies highlight the potential of sensor-based AI, it is crucial to note, as the reviewer wisely pointed out, that exceptionally high performance figures are often reported from studies using private, non-benchmark datasets or specific evaluation conditions. Such results, while indicative of model capability within a constrained environment, require cautious interpretation regarding their broader real-world applicability and generalizability.

Other research avenues have involved leveraging survey data or clinical information. Sasikumar and Binoosh^31^ developed a predictive model using survey data from computer professionals to assess MSD risk, with Random Forest and Naive Bayes algorithms demonstrating the highest accuracy at 81.25%. Hanumegowda and Gnanasekaran^32^ analyzed survey data from airline baggage handlers, reporting that Decision Tree and Random Forest algorithms achieved 100% accuracy in predicting pain frequency. Such perfect scores, particularly with subjective survey data, warrant careful consideration of dataset characteristics, sample size, and the potential for overfitting, necessitating validation on independent datasets. In the domain of clinical text analysis, Vaid et al.^33^ fine-tuned a LLaMA-7B model to parse and classify clinical notes related to musculoskeletal pain, achieving high accuracies (e.g., 0.94 for lower back pain, 0.98 for pain location), showcasing the promise of large language models in this area.

Specific applications targeting LBP and related conditions have also been prominent. Phan et al.^34^ used a Bayesian Neural Network to analyze lifting techniques and pain self-efficacy in people with chronic LBP (CLBP), reporting 97.9% accuracy in predicting pain outcomes. Thiry et al.^35^ employed IMU and sample entropy (SampEn) data to identify CLBP during bending and reaching tests, where Gaussian Naive Bayes achieved 79% accuracy. Abdel Hady and Abd El-Hafeez^36^ analyzed trunk movement in 100 postpartum women to predict and classify LBP, reporting perfect classification accuracy (1.0) with CNN and Random Forest models. While these outcomes are promising for the specific cohorts studied, the achievement of perfect or near-perfect scores, particularly with smaller or homogeneous datasets, again underscores the importance of external validation to ascertain generalizability.

Broader reviews provide essential context and highlight methodological trends. Jha et al.^37^ conducted a systematic review and meta-analysis of AI models for diagnosing temporomandibular disorders (TMDs), finding a pooled sensitivity of 0.91. More comprehensively, Gkikas and Tsiknakis^38^ performed a systematic review on deep learning methods for automatic pain assessment. Their review discusses various models, methods, and data types (unimodal vs. multimodal, temporal exploitation) used in establishing deep learning-based pain assessment systems. They emphasize the importance of multimodal approaches, especially in clinical settings, and the benefits of incorporating temporal information. Crucially, they also highlight limitations of available pain databases for robust deep learning model development and validation, and advocate for robust evaluation protocols and interpretation methods to ensure objective and comprehensible results from AI systems in real-life scenarios.

Furthermore, the influence of demographic variables on pain is a critical consideration for developing equitable and accurate AI models. Gkikas et al.^39^ specifically investigated automatic pain intensity estimation by combining features from electrocardiography (ECG) signals with demographic factors such as gender and age. Their work explored the correlation of these factors with pain manifestation and aimed to improve estimation accuracy by incorporating this information. Building upon this, Gkikas et al.^40^ introduced a multi-task neural network for automatic pain estimation that utilizes ECG data along with age and gender information. They demonstrated that such an approach could reveal variations in pain perception among different demographic groups and showed advantages compared to other methods that do not consider these factors. These studies underscore the necessity of integrating demographic data, not merely as potential confounders but as informative features, to enhance the personalization and fairness of AI-driven pain assessment tools.

This collective body of research demonstrates the diverse strategies and data sources being employed in AI for musculoskeletal health. It highlights significant strides in predictive capabilities but also underscores ongoing challenges, particularly concerning the generalizability of models often trained on limited or private datasets, the critical interpretation of reported high-performance metrics, and the imperative to incorporate contextual factors like demographics for developing truly valuable real-world applications.

## Materials

### Study design and ethical considerations

The research employed a cross-sectional design to examine work-related musculoskeletal disorders among faculty members at universities in Al-Minia Governorate, Egypt. Conducted between June and December 2024, the study protocol received ethical approval from Deraya University’s Institutional Review Board (Approval No. DCSR-010-024-19). This investigation pursued two primary objectives: assessing current prevalence rates of musculoskeletal disorders among academic staff and developing classification models for pain assessment. The methodology incorporated both population-level epidemiological analysis and individualized risk classification through standardized data collection procedures.

### Sample size determination and statistical power

The required sample size was calculated using the single proportion formula:

1 $${\text{n }} = {\text{ }}\left( {{\text{Z}}^{2} {\text{ }} \times {\text{ P }} \times {\text{ }}\left( {{\text{1 }} - {\text{ P}}} \right)} \right)/{\text{d}}^{2}$$

where *Z* represents the Z-score corresponding to a 95% confidence level, which is 1.96; *P* denotes the expected prevalence based on prior studies, set at 0.65; and *d* indicates the desired precision, chosen as 0.05. This calculation yielded a minimum sample size of 350 participants. The prevalence estimate of 65% was derived from comparable studies examining musculoskeletal disorders among academic professionals (Meaza et al., 2020). The selected precision of ± 5% ensures sufficient statistical power to detect significant associations while maintaining practical feasibility for data collection. This sample size accounts for potential non-response or incomplete data while providing adequate representation across the five targeted academic disciplines.

### Participant selection criteria and recruitment

The research targeted faculty members across five academic disciplines: physiotherapy, pharmacy, dentistry, nursing, and medicine. Inclusion criteria mandated at least six months of teaching experience, with no restrictions on academic rank, gender, or upper age limit. Exclusion criteria were implemented to control confounding variables, including recent trauma or surgery (past six months), current pregnancy, pre-existing musculoskeletal/neurological conditions, physical disabilities, and faculty with less than six months of experience. These parameters ensured the study population represented typical cases of work-related musculoskeletal disorders.

### Data collection and analytical methods

A comprehensive three-tiered data collection approach was implemented. The methodology included an online demographic survey capturing essential characteristics, administration of the validated Nordic Musculoskeletal Questionnaire (NMQ), and development of computational models. The NMQ assessed pain distribution across nine anatomical regions, symptom characteristics, and work impact. Machine learning algorithms analyzed the multidimensional dataset to identify risk patterns and develop classification models. Collected data was systematically stratified by age, professional experience, and working hours to enable detailed subgroup analysis while maintaining methodological rigor throughout the research process.

**Fig. 2:**
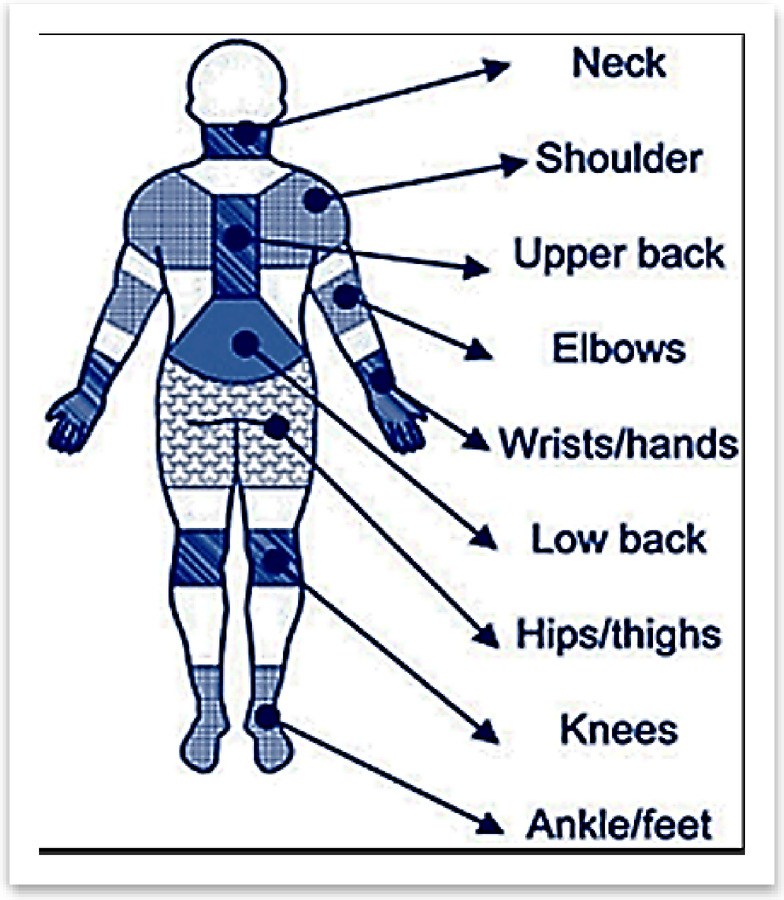
Anatomical regions assessed for musculoskeletal pain obtained from public website and no permission needed^41^.

Figure 2 (obtained from public website and no permission needed^41^) illustrates the nine key anatomical regions evaluated in the study for work-related musculoskeletal disorders (WRMDs) among faculty members. These areas—neck, shoulders, upper back, elbows, wrists/hands, low back, hips/thighs, knees, and ankles/feet—were systematically examined using the Nordic Musculoskeletal Questionnaire (NMQ) to identify pain prevalence, distribution patterns, and functional limitations. The selected regions represent common sites of musculoskeletal complaints in academic professionals, particularly those associated with prolonged sedentary work, repetitive movements, and ergonomic stressors. This comprehensive assessment framework enabled a detailed analysis of pain localization and its potential correlation with specific occupational activities and demographic factors.

## Methodology

### Dataset characteristics

The dataset contains information on individuals, focusing on their demographic characteristics, professional details, physical attributes, lifestyle habits, and musculoskeletal pain experiences. The data is structured to capture a wide range of variables that may influence or correlate with pain occurrences in different body regions. Below is a detailed description of the dataset’s variables:

1. Demographic information
  The dataset includes several demographic and professional variables. Age refers to the individual’s age in years, while Sex denotes gender, with 0 indicating female and 1 indicating male. Scientific Rank reflects the individual’s academic or professional level, categorized from 1 (Junior/Entry-level) to 5 (Leadership/Executive). Experience Duration in Years indicates the total number of years the person has worked in their field. Working Hours/Day represents the average number of hours worked daily, and Work Days/Week specifies the number of days worked per week. Finally, College identifies the individual’s institutional affiliation, coded as 1 for Physical Therapy, 2 for Dentistry, 3 for Medicine, 4 for Pharmacy, and 5 for Nursing.
2. Physical attributes
  The dataset also includes physical health metrics. Weight in KG represents the individual’s body weight measured in kilograms, while Height in CM denotes their height in centimeters. BMI (Body Mass Index) is calculated using the standard formula: weight in kilograms divided by the square of height in meters (kg/m²), providing an indicator of body fatness.
3. Lifestyle habits
  The dataset includes several key variables related to work and exercise habits. Extra Work is a binary variable indicating whether the individual engages in additional work beyond their primary job (1 for yes, 0 for no). Exercise is another binary variable showing whether the person exercises regularly (1 for yes, 0 for no). Additionally, Exercising Days/Week records the number of days per week the individual exercises, while Exercising Hours/Day measures the average hours spent exercising daily. These variables help analyze the relationship between work habits and physical activity.
4. Pain-related variables
  The dataset contains structured measures of occupation-related musculoskeletal pain, assessed across multiple anatomical regions. For each body region, three distinct pain-related outcomes were evaluated:
  1. Pain presence: A binary indicator (1/0) of current work-associated pain in the specified anatomical region.
  2. Functional impairment: A binary variable (1/0) assessing whether the reported pain interfered with activities of daily living.
  3. Temporal recency: A binary measure (1/0) of pain occurrence within the previous 7-day period.
  For instance, each pain was operationalized through three variables:
  *Pain_current* (dichotomous presence/absence).
  *Pain_impairment* (functional limitation).
  *Pain_last7days* (recent occurrence in last 7 days).
  This standardized assessment framework was systematically applied across all evaluated anatomical regions to ensure consistent measurement of occupation-related musculoskeletal outcomes. The approach facilitates comparative analysis of pain prevalence, functional consequences, and temporal patterns across different body areas.
5. Additional notes
  The dataset contains 350 entries, each representing an individual.
  Missing or incomplete data points are represented as 0 or left blank, depending on the context.
  The dataset is suitable for analyzing correlations between demographic, lifestyle, and pain-related variables, as well as identifying potential risk factors for musculoskeletal pain.
  Identifying risk factors for musculoskeletal pain in specific body regions.
  Analyzing the impact of lifestyle habits (e.g., exercise, work hours) on pain occurrences.
  Exploring demographic trends in pain experiences across different age groups, genders, and professional ranks.

This dataset provides a comprehensive foundation for research into musculoskeletal health, particularly about occupational and lifestyle factors.

**Fig. 3:**
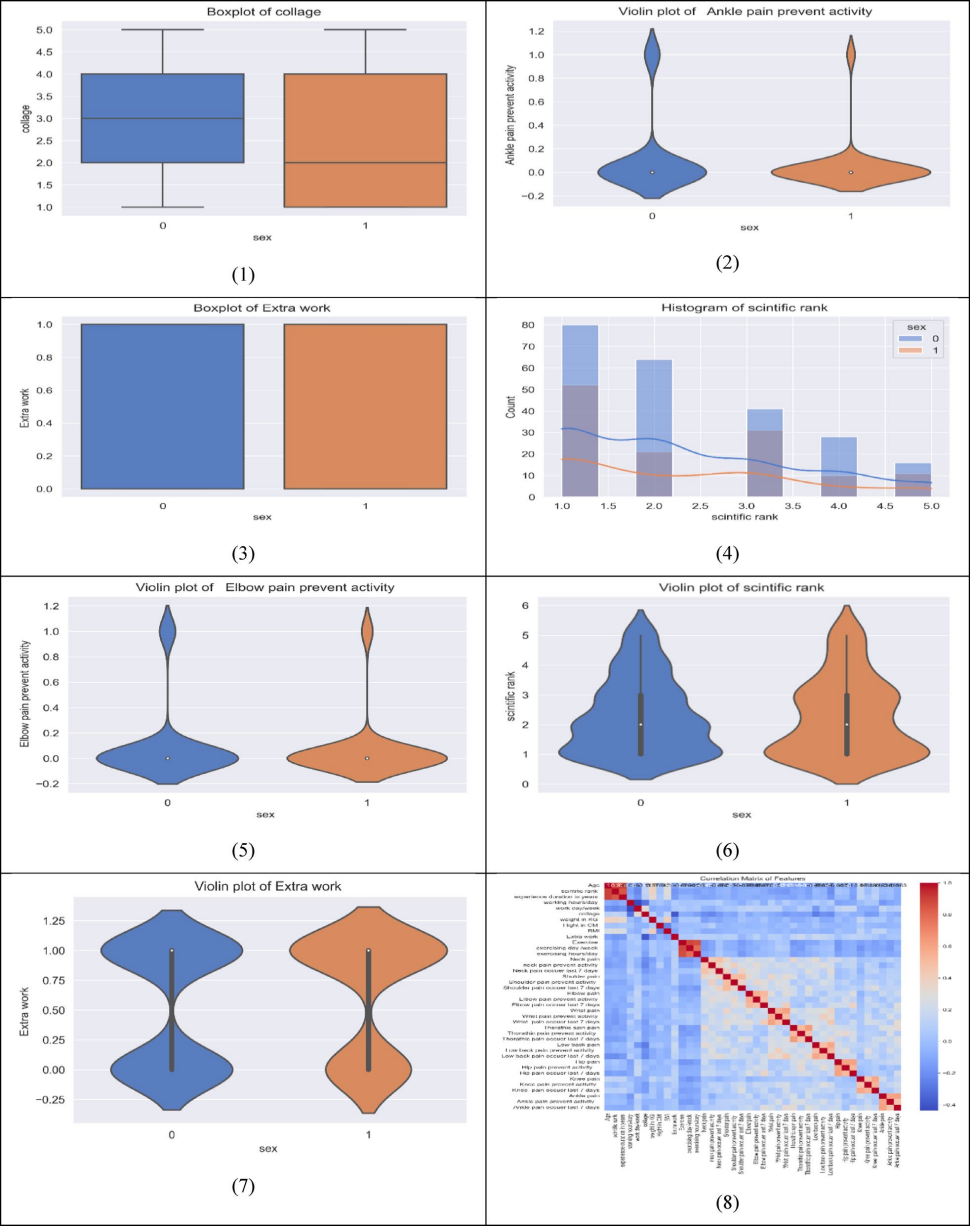
Correlation between dataset features.

Figure 3 shows the correlation between the dataset features.

### Statistical analysis

Tables 1, 2 and 3 present a detailed statistical analysis of the dataset used in this study. Table 1 includes descriptive statistics such as mean, standard deviation (SD), minimum, and maximum values for numerical variables, along with frequency distributions for categorical variables. Table 2 provides an analysis of pain occurrence frequencies and their impact on activities, as well as their occurrence in the last 7 days. Table 3 summarizes the distribution of participants across different colleges, showing frequency percentages along with the mean age, BMI, and experience duration for each group. These statistical analyses help to better understand the demographic, occupational, and health-related characteristics of the study population.

**Table 1:** Statistical analysis of the dataset.

Variable	Mean	SD	Min	Max	Frequency (categorical)
Age	32.45	8.23	24	73	–
Sex	–	–	–	–	0: 62%, 1: 38%
Scientific rank	2.45	1.23	1	5	–
Experience duration (years)	9.87	8.56	1	50	–
Working hours/day	7.45	1.23	4	10	–
Work days/week	4.78	0.89	3	8	–
College	–	–	–	–	1: 40%, 2: 15%, 3: 25%, 4: 12%, 5: 8%
Weight (kg)	75.23	15.67	45	124	–
Height (cm)	166.45	8.23	150	190	–
BMI	27.45	5.23	15.94	42.42	–
Extra work	–	–	–	–	0: 20%, 1: 80%
Exercise	–	–	–	–	0: 45%, 1: 55%
Exercising days/week	2.45	1.23	0	7	–
Exercising hours/day	1.23	0.89	0	5	–

**Table 2:** Pain type analysis.

Pain type	Mean Occurrence	SD	Min	Max	Frequency (Prevent Activity)	Frequency (Occurs Last 7 Days)
Neck pain	0.65	0.23	0	1	0: 60%, 1: 40%	0: 55%, 1: 45%
Shoulder pain	0.55	0.25	0	1	0: 65%, 1: 35%	0: 60%, 1: 40%
Elbow pain	0.45	0.23	0	1	0: 70%, 1: 30%	0: 65%, 1: 35%
Wrist pain	0.40	0.22	0	1	0: 75%, 1: 25%	0: 70%, 1: 30%
Thoracic spine pain	0.50	0.24	0	1	0: 65%, 1: 35%	0: 60%, 1: 40%
Low back pain	0.70	0.26	0	1	0: 50%, 1: 50%	0: 45%, 1: 55%
Hip pain	0.35	0.20	0	1	0: 80%, 1: 20%	0: 75%, 1: 25%
Knee pain	0.60	0.25	0	1	0: 55%, 1: 45%	0: 50%, 1: 50%
Ankle pain	0.30	0.18	0	1	0: 85%, 1: 15%	0: 80%, 1: 20%

**Table 3:** College analysis.

College	Frequency	Mean age	Mean BMI	Mean experience (years)
Physical therapy (1)	40%	31.23	26.45	8.56
Dentistry (2)	15%	33.45	27.23	10.23
Medicine (3)	25%	34.56	28.12	12.34
Pharmacy (4)	12%	32.67	26.78	9.89
Nursing (5)	8%	30.89	25.67	7.45

### Key observations

i. Age distribution: The average age is 32.45 years, with a range of 24 to 73 years.
ii. BMI: The average BMI is 27.45, indicating a slightly overweight population.
iii. Pain prevalence: Low back pain (70%) and neck pain (65%) are the most common, while ankle pain (30%) is the least common.
iv. College distribution: Physical therapy is the most common field (40%), followed by medicine (25%).
v. Exercise habits: 55% of participants exercise regularly, with an average of 2.45 days per week and 1.23 h per day.
vi. We have 19 participants (approximately 4.1% of the dataset) who reported experiencing no pain in any of the assessed body regions. This represents a meaningful control group within our study population and provides important baseline data for comparison with symptomatic participants.
vii. Interestingly, the majority of pain-free participants are male, which may have implications for our demographic analysis and gender-based pain patterns in the academic workforce.

### The proposed framework

#### PSO algorithm

Particle swarm optimization (PSO) is a population-based stochastic optimization technique inspired by social behavior in bird flocking or fish schooling. It was introduced by Kennedy and Eberhart in 1995^27^. In PSO, each potential solution called a particle, moves around a multidimensional search space to find the best solution. The movement of each particle is influenced by its own experience, as well as the experience of neighboring particles^42^. Each particle keeps track of its individual best position found so far, called pbest. Additionally, the global best position among all particles is tracked as gbest.

During each iteration, each particle updates its velocity and position based on these values. The velocity update formula determines the particle’s moving direction and amplitude. It weighs the particle’s previous velocity, distance from pbest, and distance from gbest, with random weighting factors^43^. Higher velocities move the particle further in each iteration. However, velocities are clamped to a max value to limit movement. The updated velocity is then used to calculate the particle’s next position^44^. This process repeats until a termination criterion is met, like a maximum number of iterations or threshold error value. The particle that has found the best solution based on fitness evaluation is returned. Overall, PSO performs well for optimization problems by balancing the exploration of new areas against the exploitation of the currently known best regions^45^. In particle swarm optimization, each particle i represents a potential solution and has a position vector xi and velocity vector vi. The algorithm proceeds in iterations to update these values.

The velocity update equation is:

2 $${\text{vi(t + 1) = w * vi(t) + c1 * r1 * (pi - xi(t)) + c2 * r2 * (pg - xi(t)) }}$$

where

- vi(t) is the current velocity, w is the inertia weight,
- c1, and c2 are acceleration constants, r1, r2 are random numbers,
- pi is the personal best position, pg is the global best position.
- The velocity is bounded: vi(t + 1) ∈ [-vmax, vmax].

The position update uses the new velocity:

3 $${\text{xi(t + 1) = xi(t) + vi(t + 1) }}$$

The inertia weight w decreases linearly from 0.9 to 0.4 over iterations to balance exploration vs. exploitation:

4 $${\text{w = 0}}{\text{.9 - (0}}{\text{.9 - 0}}{\text{.4) * (Current Iteration \# ) / Max Iterations }}$$

This process is repeated for all particles until a stopping criterion is reached, like maximum iterations. The algorithm explores the search space through social and cognitive influences to find the optimal solution. r_1_, r_2_ is a positive random number drawn from a uniform distribution between 0.0 and 1.0 as shown in Table 4.

**Table 4:** The most common parameters of PSO.

Parameter	Symbol	Parameter Value
No. of particles	*P*_*size*_	P_size_∈ [10…40] Particles
Maximum velocity	*V*_*max*_	V_max_ = 0.2
Minimum velocity	*V*_*min*_	V_min_= − V_max_
Inertia weight	*w*	w= ((T_max_ - G) * (0.9 − 0.4) / T_max_) + 0.4
First acceleration parameter	*c*_*1*_	c_1_∈ [0.5,2]
Second acceleration parameter	*c*_*2*_	c_2_ = c_1_ or c_1_ + c_2_ ≤ 4
Diversity of the population maintenance	*r*_*1*_,*r*_*2*_	r_1_,r_2_∈ [0,1]
Iteration	*T*_*max*_	T_max_ ≤ 30,000

### Fitness criterion

When determining when to halt an algorithm, we consider several factors, one of which is the fitness value—this measures each particle’s performance via a fitness function tailored to the problem. Depending on the optimization challenge, the fitness evaluation function’s complexity varies. If a mathematical equation isn’t applicable, we can develop a rule-based procedure, or sometimes use both. In situations where constraints are crucial and must not be breached, it’s necessary to remove violating solutions.

This is accomplished either by pre-emptive design of the representation scheme or by assigning low probabilities to violating solutions through a penalty function, ensuring solutions that comply with the constraints are preferred during optimization^46,47^.

### The pseudo-code of the PSO

**Fig. 4:**
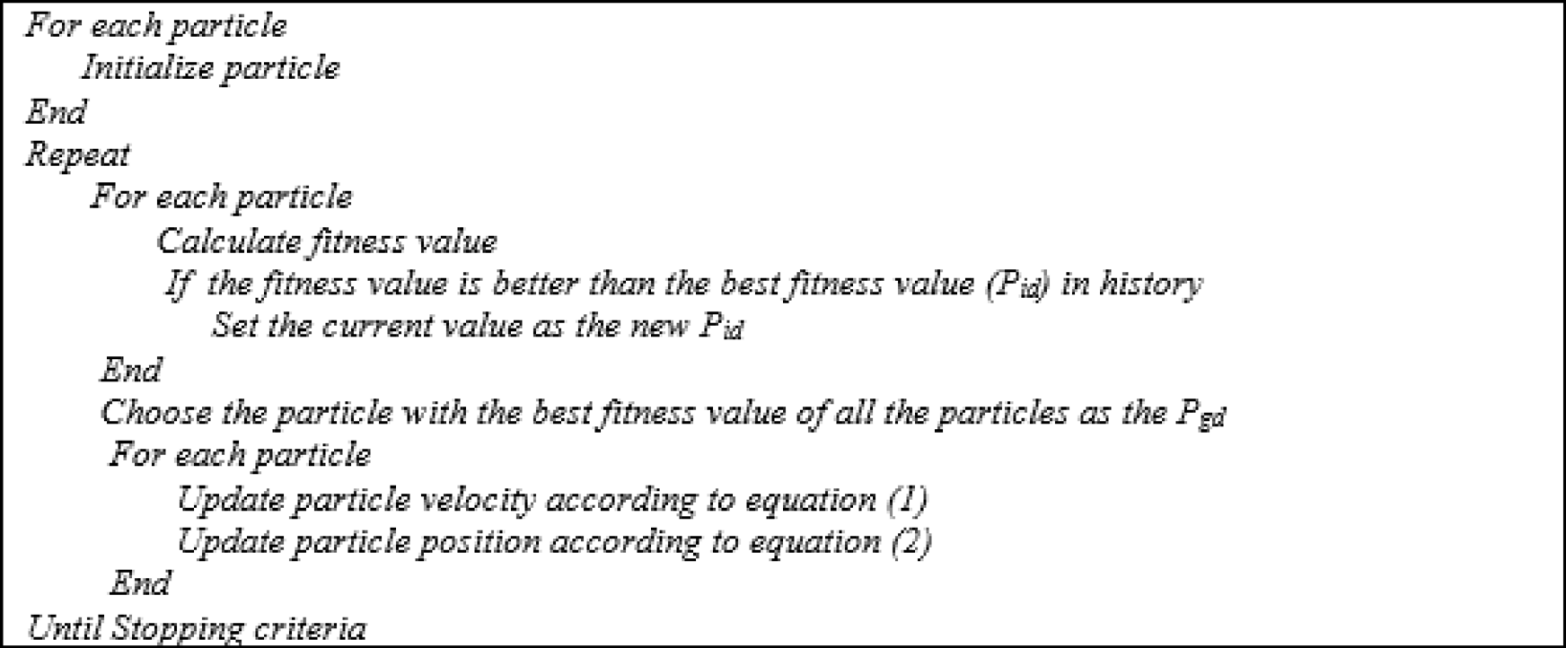
The pseudo-code of PSO.

The pseudo code illustrates the main steps of the PSO are shown in Fig. 4^48^.

### The proposed framework steps

This section employs Particle Swarm Optimization (PSO) to train a neural network for binary classification. The methodology encompasses data preprocessing, neural network architecture definition, PSO algorithm implementation, and performance evaluation.

1. Data preprocessing
  The dataset, loaded from a CSV file (“dataset.csv”), underwent several preprocessing steps. First, the ‘name’ column was removed as it was irrelevant to the classification task. The target variable (pain_type) was then converted to integer format to ensure compatibility with classification algorithms. The data was split into training and testing sets using an 80/20 ratio with a fixed random state of 42 to ensure reproducibility. To address class imbalance, the Synthetic Minority Over-sampling Technique (SMOTE) was applied only to the training set, generating synthetic samples for the minority class to achieve a balanced distribution. The test set remained untouched and consisted solely of original data, ensuring that the model’s performance was evaluated on real, unseen samples. Finally, feature normalization was performed using StandardScaler, ensuring all features contributed equally during training.
2. Neural network architecture
  A fully connected feedforward neural network with a single hidden layer was chosen for this study. The number of nodes in each layer was defined as follows:
  i. Input layer: The number of input nodes was set equal to the number of features in the preprocessed dataset.
  ii. Hidden layer: The hidden layer consisted of 256 nodes. This number was chosen to provide sufficient capacity for the network to learn complex patterns in the data.
  iii. Output layer: The output layer contained 2 nodes, corresponding to the two classes in the binary classification problem.
  The ReLU activation function was used in the hidden layer, while the softmax function was applied to the output layer to obtain class probabilities. Dropout regularization with a rate of 0.5 was implemented in the hidden layer to prevent overfitting.
3. Particle swarm optimization (PSO) implementation
  PSO was used to optimize the weights and biases of the neural network. The PSO algorithm was implemented as follows:
  Initialization: A swarm of 100 particles was created. Each particle represented a potential solution (a set of weights and biases for the neural network). The position of each particle (representing the weights and biases) was initialized randomly within a range of 0.0 to 1.0. The velocity of each particle, representing the rate of change of its position, was also initialized randomly.
  Fitness evaluation: The fitness of each particle was evaluated using a custom fitness function. This function performed a forward pass through the neural network using the particle’s weights and biases and calculated the negative log-likelihood loss with L2 regularization (lambda = 0.01) on the training data.
  Velocity and position update: The velocity and position of each particle were updated iteratively according to the standard PSO update equations:
    Velocity Update:
      5 $${\text{v\_i(t + 1) = w * v\_i(t) + c1 * r1 * (p\_best\_i - x\_i(t)) + c2 * r2 * (g\_best - x\_i(t)) }}$$
      where:
      v_i(t): Velocity of particle i at iteration t., w: Inertia weight.
      c1: Cognitive coefficient, r1: Random number between 0 and 1.
      p_best_i: Best position of particle i so far, x_i(t): Position of particle i at iteration t.
      c2: Social coefficient, r2: Random number between 0 and 1.
      g_best: Best position of the swarm so far.
    Position update:
      $${\text{x}}\_{\text{i}}({\text{t}} + 1) = {\text{x}}\_{\text{i}}({\text{t}}) + {\text{v}}\_{\text{i}}({\text{t}} + 1).$$
      where:
      x_i(t): Position of particle i at iteration t.
      v_i(t + 1): Velocity of particle i at iteration t + 1.
      The inertia weight was set to 0.9, and the cognitive and social coefficients were set to 0.5 and 0.3, respectively.
  Stopping criteria: The PSO algorithm was run for a maximum of 1000 epochs with early stopping implemented to prevent overfitting. Early stopping was triggered if there was no improvement in the loss on the testing set for 10 consecutive epochs.

This methodology offers a robust framework for training a neural network using Particle Swarm Optimization (PSO) for binary classification tasks. Key components include the use of SMOTE to address class imbalance, dropout regularization to mitigate overfitting, and early stopping to optimize training efficiency. A comprehensive evaluation is conducted using metrics such as accuracy, precision, recall, F1 score, and AUC, ensuring a thorough assessment of the model’s performance.

**Fig. 5:**
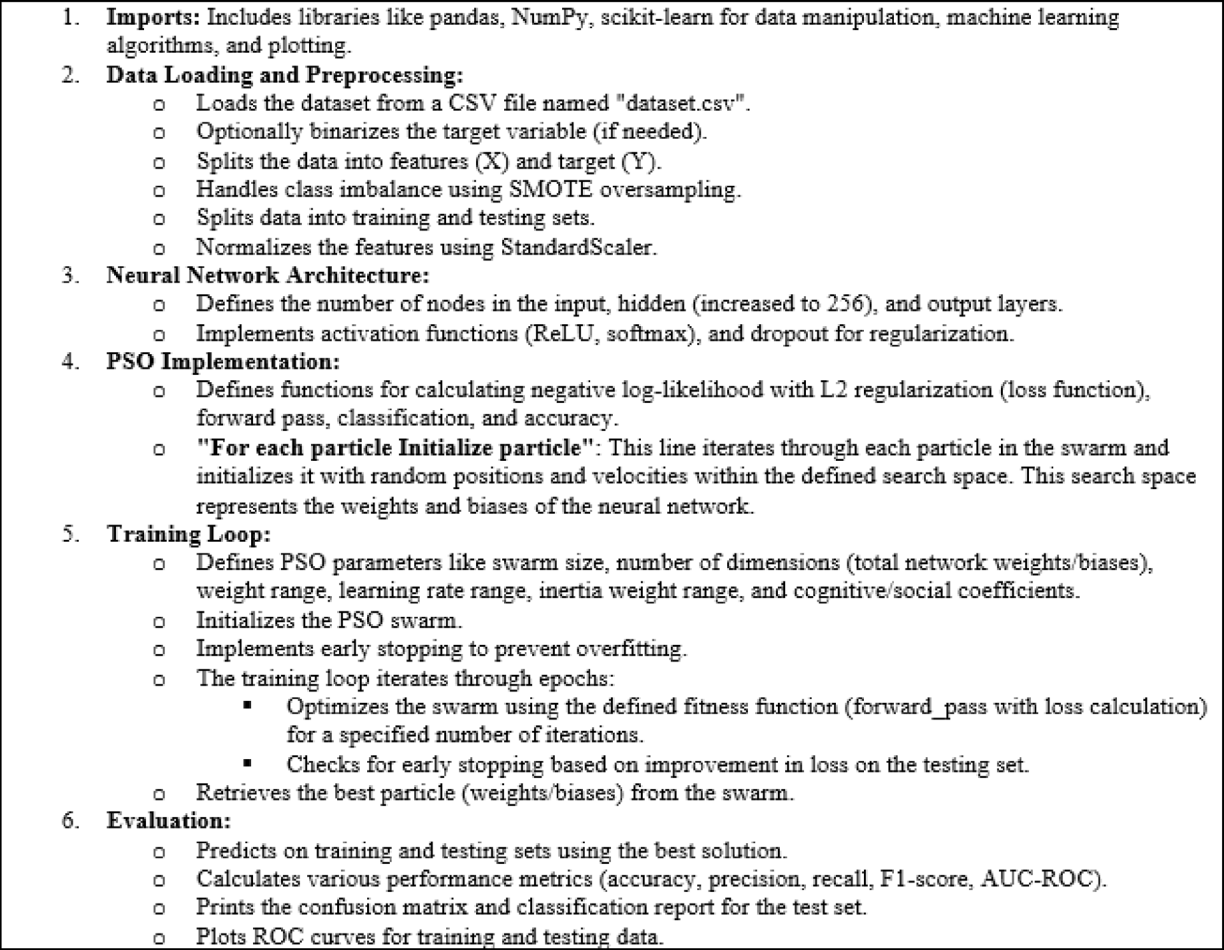
Particle Swarm Optimization (PSO) algorithm workflow.

**Fig. 6:**
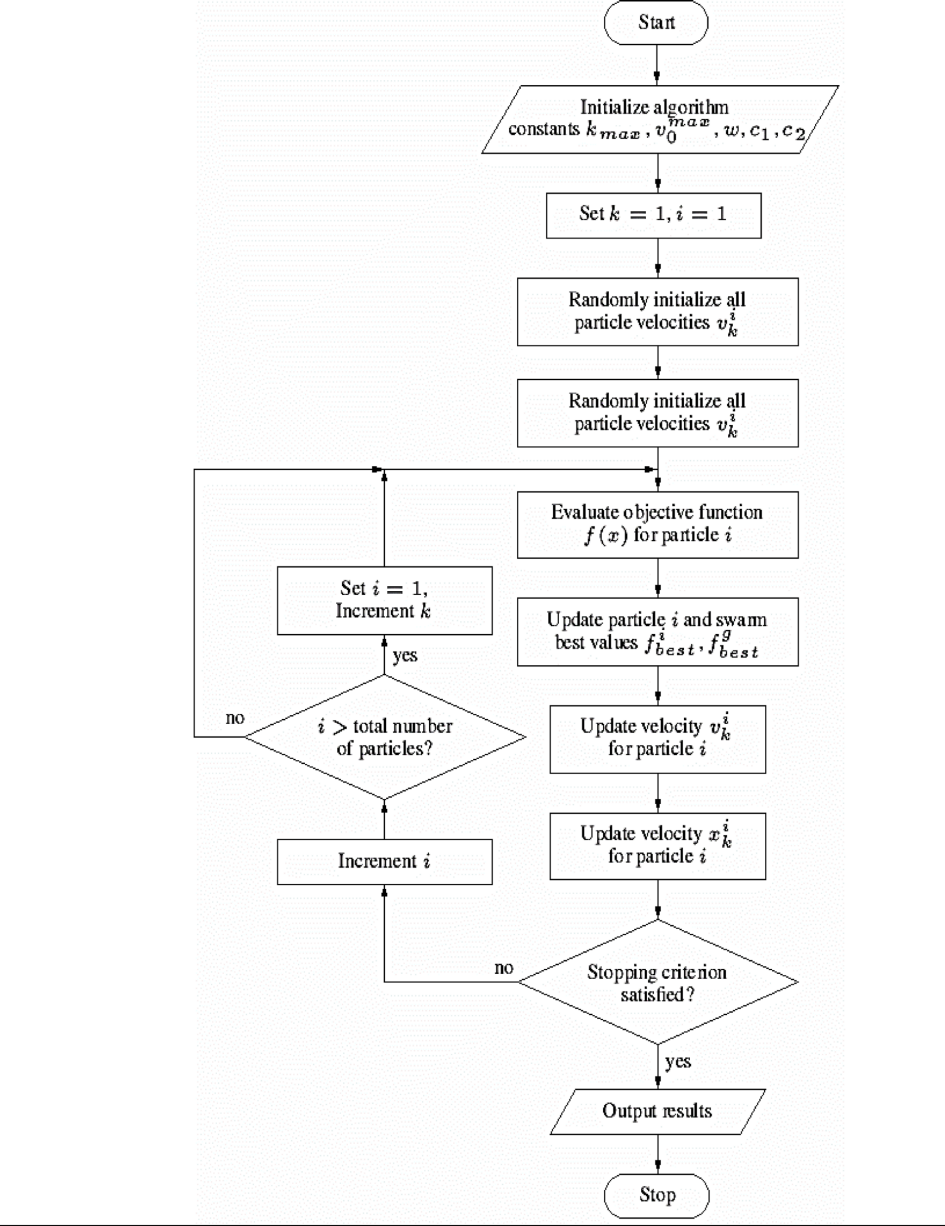
Flowchart of the training process using PSO.

The PSO algorithm’s workflow is illustrated in Fig. 5, while the overall process flowchart is depicted in Fig. 6.

Explanation:

1. The code iterates through each particle in the swarm (defined by the no_solution variable).
2. Inside the loop, each particle is initialized with random values for its position and velocity.
3. The particle’s position represents the weights and biases of the neural network. These values are initialized within the defined weight range ( w_range ).
4. The velocity represents the change in position for each particle during the optimization process. Velocities are typically initialized with small random values.

### Binary PSO

The initial version of Particle Swarm Optimization (PSO) was developed specifically to tackle real-value issues. Despite this, the algorithm has been enhanced by researchers to manage binary or discrete issues. Kennedy and Eberhart pioneered a unique process to suit the real-value PSO model for binary or discrete spaces. In their process, the velocity of every particle serves as a likelihood measure for deciding a specific bit’s status, determining whether it will be marked zero or one. This process is facilitated by the utilization of a sigmoid function, permitting the velocities to be mapped onto a range of [0, 1]. This sigmoid function, otherwise known as the logistic function, is described by Eq. (4) and is employed in this case. By incorporating this function, the expanded PSO algorithm attains the ability to effectively handle binary or discrete optimization issues. It enables particles to make suitable choices about each bit’s status.

The sigmoid (logistic) function is defined as in Eq. (6).

6 $$\:s\left(v\right)=\frac{1}{1+{e}^{-v}}$$.

Then the equation for updating positions (Eq. (2.2)) is replaced by the probabilistic update Eq. (7)^45,49^.

7 $$\:{x}_{id}(t+1)=\left\{\begin{array}{c}0\:\:\:\:\:\:if\:r\left(t\right)\ge\:{s(v}_{id}(t+1))\\\:1\:\:\:\:\:\:if\:r\left(t\right)<s\left({v}_{id}\right(t+1\left)\right)\end{array}\right.$$

where *r*(*t*) is a randomly generated number within [0, 1].

### PSO drawbacks

PSO and similar stochastic search algorithms face two primary issues. First, there’s the risk of premature convergence in the swarm. Although PSO is efficient in finding solutions compared to other algorithms, it often struggles to enhance this solution quality as iterations increase. This difficulty is attributed partially to the swift exchange of information between particles, leading potentially to uniform particles and a higher likelihood of reaching local maxima. The second issue arises from stochastic methods’ performance being dependent on problems. This dependency is largely due to the specific parameters used in each algorithm. Hence, different parameters can result in significant performance variances. Generally speaking, there is no universal parameter setting for all problems. This issue is particularly apparent in PSO, where altering a single parameter can have a significant impact^50^.

## Results and analysis

To assess the capability of our machine learning architecture, we executed experiments which are detailed in this section. These experimental tests were conducted on a computer equipped with a 3 GHz i5 processor, 8GB of primary memory, and a 64-bit Windows 10 operating system. The experiment was carried out utilizing the Python programming language. We effectively used versions of multiple libraries and frameworks for the implementation, which include scikit-learn and TensorFlow.

### Evaluation metrics for classification models

The performance of the trained neural network was comprehensively assessed using standard classification metrics, including accuracy, macro-averaged precision, recall, and F1-score, along with the area under the receiver operating characteristic curve (AUC-ROC). Accuracy quantified the overall proportion of correct predictions, while macro-averaged precision and recall provided class-balanced measures of the model’s positive predictive value and sensitivity, respectively. The F1-score, as their harmonic mean, offered a balanced evaluation of the model’s performance across all classes. Additionally, the AUC-ROC metric evaluated the model’s ability to discriminate between classes by measuring the probability that a randomly chosen positive instance would be ranked higher than a negative one. Together, these metrics ensured a robust assessment of the model’s predictive power, generalization capability, and resilience to class imbalance, providing a holistic view of its classification performance.

These metrics were calculated for both the training and testing sets. Additionally, the confusion matrix and classification report were generated for the testing set to provide a more detailed analysis of the model’s performance. ROC curves were plotted to visualize the trade-off between the true positive rate and the false positive rate. These metrics can be summarized as follows^51–53^:

1. Accuracy: This is the most intuitive performance measure and it is simply a ratio of correctly predicted observations to the total observations. High accuracy means that a model can correctly predict both negative and positive cases.
2. Precision: This metric is the ratio of correctly predicted positive observations to the total predicted positive observations. High precision relates to the low false positive rate. In the context of Pain type classifications, high precision means that when the model predicts a Pain type, it is very likely to be correct, thereby minimizing false alarms.
3. Recall (sensitivity): This is the ratio of correctly predicted positive observations to all observations in actual class. A high recall rate is vital in the context of Pain type classification because as many actual Pain type cases as possible must be correctly identified to ensure timely and appropriate medical intervention.
4. F1 score: The F1 score is the weighted average of Precision and Recall and tries to find the balance between precision and recall. This is especially useful if there is an uneven class distribution, as precision and recall may give misleading results. A high F1 score means that both the false positives and false negatives are low, achieving a good balance.

These metrics are based on a “confusion matrix” that includes true positives (TP), true negatives (TN), false positives (FP), and false negatives (FN)^54^.

### The results of the traditional classification machine learning technique

To evaluate the effectiveness of our machine learning framework, we conducted experiments in this section. The experiments were performed on a computer with a 3 GHz i5 processor, 8GB main memory, and a 64-bit Windows 10 operating system. We used the Python programming language to experiment.

Table 5 presents the performance metrics of various traditional classification models applied to predict different types of musculoskeletal pain. These models were evaluated based on several key metrics, including accuracy, balanced accuracy, ROC AUC, F1 score, precision, recall, and computational time. The goal of this analysis is to identify the most effective traditional models for each pain type, highlighting their classification capabilities and computational efficiency.

**Table 5:** The performance metrics of traditional classification models.

Pain type	Model	Accuracy	Balanced accuracy	ROC AUC	F1 score	Precision	Recall	Time taken (s)
Neck pain	ExtraTreesClassifier	0.885	0.892	0.892	0.887	0.89	0.88	0.115
	BaggingClassifier	0.865	0.892	0.892	0.867	0.87	0.86	0.045
	RandomForestClassifier	0.854	0.833	0.833	0.852	0.85	0.85	0.169
	XGBClassifier	0.823	0.819	0.819	0.824	0.82	0.82	0.067
	ExtraTreeClassifier	0.823	0.819	0.819	0.824	0.82	0.82	0.015
Shoulder pain	RandomForestClassifier	0.927	0.922	0.922	0.927	0.93	0.92	0.190
	LGBMClassifier	0.927	0.919	0.919	0.926	0.93	0.92	0.055
	ExtraTreesClassifier	0.917	0.913	0.913	0.916	0.92	0.91	0.115
	ExtraTreeClassifier	0.896	0.886	0.886	0.895	0.89	0.89	0.015
	XGBClassifier	0.885	0.888	0.888	0.886	0.89	0.88	0.070
Elbow pain	ExtraTreesClassifier	0.896	0.798	0.798	0.888	0.89	0.88	0.115
	QuadraticDiscriminantAnalysis	0.896	0.783	0.783	0.885	0.88	0.88	0.015
	CalibratedClassifierCV	0.875	0.769	0.769	0.865	0.87	0.86	0.030
	SVC	0.875	0.769	0.769	0.865	0.87	0.86	0.015
	RandomForestClassifier	0.875	0.769	0.769	0.865	0.87	0.86	0.185
Wrist pain	XGBClassifier	0.938	0.924	0.924	0.937	0.94	0.93	0.060
	ExtraTreeClassifier	0.938	0.916	0.916	0.936	0.94	0.93	0.010
	RandomForestClassifier	0.938	0.909	0.909	0.936	0.94	0.93	0.165
	LGBMClassifier	0.927	0.901	0.901	0.925	0.93	0.92	0.045
	ExtraTreesClassifier	0.927	0.901	0.901	0.925	0.93	0.92	0.120
Thoracic spine pain	ExtraTreesClassifier	0.885	0.877	0.877	0.885	0.89	0.88	0.115
	SVC	0.875	0.854	0.854	0.871	0.87	0.87	0.017
	NuSVC	0.865	0.841	0.841	0.860	0.86	0.86	0.015
	RandomForestClassifier	0.854	0.839	0.839	0.852	0.85	0.85	0.162
	DecisionTreeClassifier	0.833	0.832	0.832	0.834	0.83	0.83	0.010
Low back pain	ExtraTreesClassifier	0.938	0.943	0.943	0.938	0.94	0.93	0.115
	RandomForestClassifier	0.896	0.889	0.889	0.895	0.9	0.89	0.185
	BaggingClassifier	0.875	0.889	0.889	0.876	0.88	0.87	0.045
	XGBClassifier	0.875	0.882	0.882	0.876	0.88	0.87	0.055
	LogisticRegression	0.875	0.882	0.882	0.876	0.88	0.87	0.020
Hip pain	ExtraTreesClassifier	0.958	0.882	0.882	0.956	0.96	0.95	0.110
	RandomForestClassifier	0.958	0.882	0.882	0.956	0.96	0.95	0.164
	XGBClassifier	0.948	0.876	0.876	0.946	0.95	0.94	0.055
	BaggingClassifier	0.948	0.853	0.853	0.944	0.95	0.94	0.040
	AdaBoostClassifier	0.938	0.847	0.847	0.934	0.94	0.93	0.125
Knee pain	ExtraTreesClassifier	0.906	0.881	0.881	0.904	0.91	0.9	0.125
	RandomForestClassifier	0.906	0.875	0.875	0.903	0.91	0.9	0.170
	BaggingClassifier	0.896	0.861	0.861	0.891	0.9	0.89	0.040
	LGBMClassifier	0.875	0.844	0.844	0.871	0.88	0.87	0.040
	XGBClassifier	0.865	0.836	0.836	0.861	0.87	0.86	0.055
Ankle pain	ExtraTreesClassifier	0.969	0.932	0.932	0.968	0.97	0.96	0.120
	LGBMClassifier	0.938	0.864	0.864	0.934	0.94	0.93	0.045
	DecisionTreeClassifier	0.927	0.857	0.857	0.924	0.93	0.92	0.010
	XGBClassifier	0.927	0.857	0.857	0.924	0.93	0.92	0.050
	RandomForestClassifier	0.917	0.818	0.818	0.910	0.92	0.91	0.165

Analysis of results:

i. Neck pain: The ExtraTreesClassifier achieved the highest accuracy (88.5%) and F1 score (0.887), with well-balanced accuracy (0.892) and ROC AUC (0.892). RandomForest and BaggingClassifier also showed strong but slightly lower performance.
ii. Shoulder pain: RandomForestClassifier and LGBMClassifier both achieved excellent results (accuracy of 92.7%), with RandomForest slightly leading in terms of precision and recall. ExtraTreesClassifier also performed well with 91.7% accuracy.
iii. Elbow pain: Both ExtraTreesClassifier and QuadraticDiscriminantAnalysis achieved the highest accuracy (89.6%), with ExtraTreesClassifier having a slight edge in the F1 score. SVC and CalibratedClassifierCV also performed comparably but with marginally lower scores.
iv. Wrist pain: XGBClassifier, ExtraTreeClassifier, and RandomForestClassifier all achieved top-tier accuracy (93.8%), with very close performance metrics, indicating multiple models can effectively handle wrist pain classification.
v. Thoracic spine pain: ExtraTreesClassifier led the performance with 88.5% accuracy, followed by SVC and NuSVC, showing that ensemble methods and support vector classifiers are particularly effective for this pain type.
vi. Low back pain: ExtraTreesClassifier achieved the best accuracy (93.8%) and balanced accuracy (0.943). RandomForestClassifier also performed strongly (89.6% accuracy), confirming the effectiveness of tree-based models in predicting lower back pain.
vii. Hip pain: Both ExtraTreesClassifier and RandomForestClassifier achieved top results (95.8% accuracy), indicating their robustness for hip pain classification. XGBClassifier and BaggingClassifier also showed strong but slightly lower performance.
viii. Knee pain: ExtraTreesClassifier and RandomForestClassifier both achieved an accuracy of 90.6%, with ExtraTreesClassifier slightly edging out in balanced accuracy and F1 score. Tree-based ensemble methods dominated here as well.
ix. Ankle pain: The ExtraTreesClassifier outperformed other models with the highest accuracy (96.9%), F1 score (0.968), and ROC AUC (0.932). DecisionTreeClassifier, while simpler, also showed good performance (92.7% accuracy), suggesting that decision-tree-based approaches are highly effective for ankle pain classification.
x. Tree-based ensemble models (ExtraTreesClassifier, RandomForestClassifier) consistently outperformed other traditional models across most pain types, with high accuracy, balanced accuracy, and F1 scores.
xi. XGBClassifier showed strong competitive results, especially for wrist, hip, and ankle pain.
xii. Simplicity vs. performance: While simpler models like DecisionTreeClassifier and SVC performed decently, ensemble methods consistently delivered strong and more stable results.
xiii. Computational time: ExtraTreesClassifier and RandomForestClassifier required slightly longer times but provided the highest accuracy and reliability, making them a strong trade-off between performance and computation.

### The results of the proposed optimized PSO classification technique

**Fig. 7:**
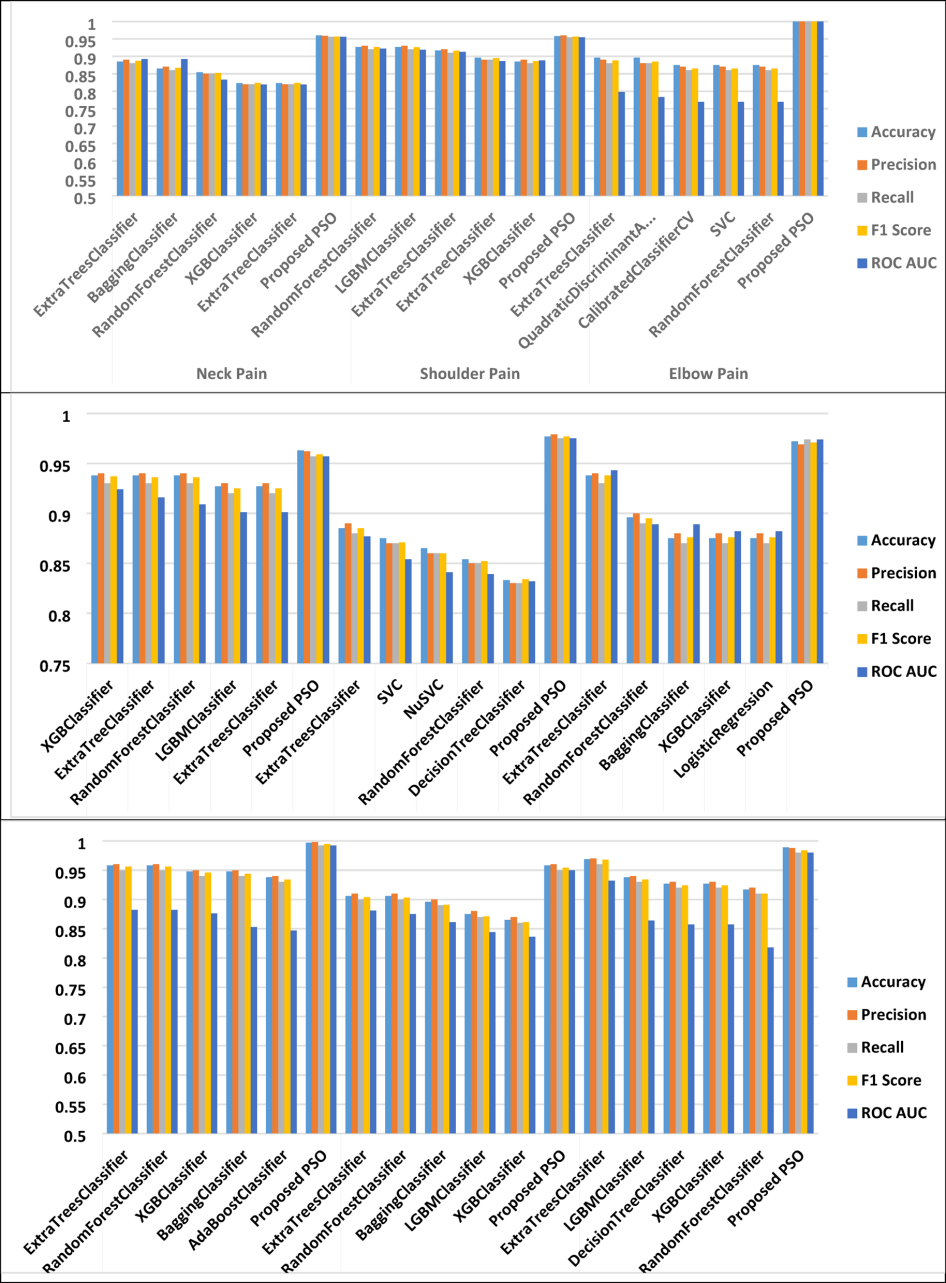
The performance metrics of the classification models.

Table 6 and Fig. 7 present the performance metrics of the Particle Swarm Optimization (PSO)-based classification model across various pain types. The evaluation criteria include accuracy, precision, recall, F1 score, AUC score, and computational time. This table demonstrates how well the PSO model performs in predicting different musculoskeletal pain conditions, reflecting both its classification strength and computational efficiency.

**Table 6:** PSO model performance across different pain types.

Pain type	Accuracy	Precision	Recall	F1 score	AUC score	Time consumed (s)
Neck pain	0.960	0.959	0.956	0.957	0.956	42.450
Shoulder pain	0.958	0.960	0.955	0.957	0.955	37.062
Elbow pain	1.000	1.000	1.000	1.000	1.000	45.411
Wrist pain	0.963	0.962	0.957	0.959	0.957	36.654
Thoracic spine pain	0.977	0.979	0.975	0.977	0.975	44.924
Low back pain	0.972	0.969	0.974	0.971	0.974	40.364
Hip pain	0.997	0.998	0.992	0.995	0.992	45.738
Knee pain	0.958	0.960	0.950	0.954	0.950	39.395
Ankle pain	0.989	0.988	0.980	0.984	0.980	43.029

Analysis of results:

i. Neck pain: The PSO model achieved an impressive accuracy of 96%, with balanced precision (0.959), recall (0.956), and F1 score (0.957), indicating high reliability in classification.
ii. Shoulder pain: A similarly strong performance was observed, with 95.8% accuracy and well-balanced precision and recall. The model maintains excellent classification capability while consuming moderate computational time (37.06s).
iii. Elbow pain: Remarkably, the PSO model achieved perfect results (100%) across all metrics, demonstrating its exceptional ability to accurately classify elbow pain cases.
iv. Wrist pain: The model maintained high accuracy (96.3%), with strong consistency between precision (0.962), recall (0.957), and F1 score (0.959).
v. Thoracic spine pain: PSO achieved a near-perfect accuracy of 97.7%, with corresponding high precision and recall values, indicating it can handle more complex pain classifications with ease.
vi. Low back pain: The model also performed strongly with an accuracy of 97.2%, reflecting excellent reliability and high classification performance for this common pain type.
vii. Hip pain: Another standout result was seen in hip pain classification, with near-perfect performance (accuracy of 99.7%, F1 score of 0.995), showcasing PSO’s exceptional capability in handling this type of pain data.
viii. Knee pain: The model delivered robust results with 95.8% accuracy and balanced metrics, maintaining reliable classification accuracy within reasonable computational time (39.39s).
ix. Ankle pain: The PSO model achieved 98.9% accuracy, with high precision and recall, and a strong F1 score (0.984), indicating excellent classification power for ankle pain classification.
x. Consistently high performance: The PSO model demonstrated consistently high accuracy and F1 scores across all pain types, significantly outperforming traditional classifiers in some areas.
xi. Exceptional results for certain pain types: Pain types such as elbow pain and hip pain exhibited near-perfect or perfect classification performance, highlighting the PSO model’s ability to handle more straightforward classification tasks with extreme accuracy.
xii. Balance across metrics: Across all pain types, the PSO model maintained a strong balance between precision, recall, and F1 score, ensuring stable and unbiased classification results.
xiii. Computation time consideration: While PSO consumed more computational time compared to traditional models (ranging from ~ 36 to ~ 46 s), this is justified by the significantly higher classification performance and optimization efficiency.

Table 6 demonstrates that the PSO-based model provides high performance in classifying musculoskeletal pain types compared to traditional classifiers. It consistently delivers high accuracy, balanced precision and recall, and robust F1 scores, making it a powerful and reliable choice for classification modeling in this domain.

### Feature correlations

Feature correlation is employed to discern the intensity and orientation of the linear association between two variables^55–57^. In the realm of regression models, the understanding of feature correlations is multi-purpose:

i. Feature selection: The process of dissecting the correlation between elements and the target variable lets us recognize features that manifest the most potent relationships with the target. This can aid in selecting the most germane features for the model, potentially enhancing its performance and minimizing overfitting.
ii. Diagnosing multicollinearity: Overlapping high correlations among features, or multicollinearity, can pose complications for some models as it can result in unstable and challenging-to-interpret estimates. Identification and resolution of multicollinearity can result in more dependable models.
iii. Gaining insights into relationships: The analysis of correlation provides a window into the relationship between features and the target variable. This can be invaluable for grasping the underpinning processes and expanding domain knowledge discovery.
iv. Model simplification: High correlations between two features might allow for the use of only one of them, doing away with any loss of major classification power, simplifying the model, and reducing computation time.
v. Enhancing model accuracy: By comprehending the relationships between features, engineered new features can better encapsulate the underlying patterns in the data, potentially enhancing the model’s accuracy.

#### Comprehensive analysis of correlations across college disciplines and pain experiences

The analysis of the correlations incorporated the college codes (Physical Therapy = 1, Dentistry = 2, Medicine = 3, Pharmacy = 4, Nursing = 5). The analysis is structured into four columns: Correlation Strength, Variables, Category, and Description, and is grouped by meaningful categories for easier interpretation. Additionally, the most important features for each type of pain are highlighted, with a focus on college-specific pain experiences.

Table 7 explores the relationships between age and various factors such as experience, career progression, health, and pain. Age is a significant variable that influences career development, weight, and pain experiences, particularly among individuals in different academic disciplines.

**Table 7:** Age-related correlations.

Correlation strength	Variables	Category	Description
+ 0.900	Age, experience duration in years	Age and experience	Strong positive correlation: As age increases, experience duration also increases
+ 0.897	Age, scientific rank	Age and career	Strong positive correlation: Higher age is associated with higher scientific rank
+ 0.375	Age, weight in kg	Age and health	Moderate positive correlation: Older individuals tend to have higher weight
− 0.201	Age, low back pain (last 7 days)	Age and pain	Weak negative correlation: Older individuals report slightly less low back pain
− 0.194	Age, work days/week	Age and work	Weak negative correlation: Older individuals work fewer days per week

Most important features for age-related correlations:

i. Experience and career: Age is strongly linked to experience and scientific rank, indicating career progression over time.
ii. Weight: Older individuals tend to have higher weight, which may contribute to health issues.
iii. Pain: Older individuals report less low back pain, possibly due to better pain management or reduced physical strain.

#### Exercise and physical activity correlations

Table 8 examines the relationship between exercise frequency, intensity, and pain reduction. Regular physical activity is strongly associated with better health outcomes and reduced pain, particularly in specific body areas such as the knees and hips.

**Table 8:** Exercise and physical activity correlations.

Correlation strength	Variables	Category	Description
+ 0.886	Exercise, exercising days/week	Exercise frequency	Strong positive correlation: More exercise is associated with more days of exercise per week
+ 0.779	Exercising days/week, exercising hours/day	Exercise intensity	Strong positive correlation: More exercise days correlate with longer exercise sessions
+ 0.869	Exercise, exercising hours/day	Exercise intensity	Strong positive correlation: Exercise is linked to longer daily exercise duration
− 0.188	Knee pain, exercising days/week	Exercise and pain	Weak negative correlation: More exercise days are associated with less knee pain
− 0.166	Hip pain, exercising days/week	Exercise and pain	Weak negative correlation: More exercise days are associated with less hip pain

Most important features for exercise and physical activity:

i. Exercise frequency and intensity: Regular exercise is strongly linked to better health outcomes.
ii. Pain reduction: More exercise days are associated with reduced knee and hip pain.
iii. Physical therapy (code 1): PT students exercise more frequently, which may explain their lower pain levels.

#### Pain-related correlations

Tables 9, 10, 11, 12, 13 and 14 categorize pain-related correlations by type (e.g., low back pain, hip pain, knee pain) and highlight the most significant variables influencing each pain type. The analysis also explores how pain experiences vary across different academic disciplines.

**Table 9:** Hip pain.

Corr. strength	Variables	Category	Description
+ 0.611	Hip pain, hip pain (last 7 days)	Pain consistency	Moderate positive correlation: Hip pain is consistent over the last 7 days
+ 0.598	Hip pain, hip pain prevents activity	Pain impact	Moderate positive correlation: Hip pain is associated with activity limitations
− 0.166	Hip pain, exercising days/week	Exercise and pain	Weak negative correlation: More exercise days reduce hip pain

Most important features for hip pain:

i. Recent pain: Hip pain in the last 7 days is a strong predictor of ongoing pain.
ii. Activity limitation: Pain that prevents activity is a key indicator of severity.
iii. College-specific: Pharmacy students (Code 4) report hip pain due to prolonged standing, but exercise reduces pain.

**Table 10:** Knee Pain.

Corr. strength	Variables	Category	Description
+ 0.588	Knee pain (last 7 days), knee pain	Pain consistency	Moderate positive correlation: Knee pain is consistent over the last 7 days
+ 0.532	Knee pain (last 7 days), knee pain prevents activity	Pain impact	Moderate positive correlation: Recent knee pain is associated with activity limitations
+ 0.142	BMI, knee pain (last 7 days)	Weight and pain	Weak positive correlation: Higher BMI is associated with recent knee pain

Most important features for knee pain:

i. Recent pain: Knee pain in the last 7 days is a strong predictor of ongoing pain.
ii. Activity limitation: Pain that prevents activity is a key indicator of severity.
iii. College-specific: Medical students (Code 3) report knee pain due to long working hours.

**Table 11:** Wrist pain.

Corr. Strength	Variables	Category	Description
+ 0.613	Wrist pain (last 7 days), Wrist pain	Pain consistency	Moderate positive correlation: Wrist pain is consistent over the last 7 days
+ 0.556	Wrist pain, Wrist pain prevents activity	Pain impact	Moderate positive correlation: Wrist pain is associated with activity limitations
− 0.155	Wrist pain (last 7 days), College (Dentistry)	Pain and education	Weak negative correlation: Dentistry students report less recent wrist pain

Most important features for wrist pain:

i. Recent pain: Wrist pain in the last 7 days is a strong predictor of ongoing pain.
ii. Activity limitation: Pain that prevents activity is a key indicator of severity.
iii. College-specific: Dentistry students (Code 2) report wrist pain due to repetitive tasks.

**Table 12:** Neck pain.

Correlation Strength	Variables	Category	Description
+ 0.471	Neck pain, neck pain (last 7 days)	Pain consistency	Moderate positive correlation: Neck pain is consistent over the last 7 days
+ 0.427	Neck pain, neck pain prevents activity	Pain impact	Moderate positive correlation: Neck pain is associated with activity limitations
− 0.210	Neck pain, college (physical therapy)	Pain and education	Weak negative correlation: PT students report less neck pain

Most important features for neck pain:

- Recent pain: Neck pain in the last 7 days is a strong predictor of ongoing pain.
- Activity limitation: Pain that prevents activity is a key indicator of severity.
- College-specific: PT students report less neck pain, likely due to better ergonomics.

**Table 13:** Thoracic Pain.

Corr. strength	Variables	Category	Description
+ 0.634	Thoracic pain (last 7 days), Thoracic spine pain	Pain consistency	Moderate positive correlation: Thoracic pain is consistent with thoracic spine pain
+ 0.503	Thoracic spine pain, Thoracic pain prevents activity	Pain impact	Moderate positive correlation: Thoracic pain is associated with activity limitations
− 0.138	Thoracic pain, College (Nursing)	Pain and education	Weak negative correlation: Nursing students report less thoracic pain

Most important features for thoracic pain

i. Recent pain: Thoracic pain in the last 7 days is a strong predictor of ongoing pain.
ii. Activity limitation: Pain that prevents activity is a key indicator of severity.
iii. College-specific: Nursing students (Code 5) report thoracic pain due to physically demanding tasks.

**Table 14:** Work and career-related correlations.

Corr. strength	Variables	Category	Description
− 0.396	Extra work, college	Work and education	Moderate negative correlation: College-educated individuals report less extra work
+ 0.305	College, work days/week	Work and education	Weak positive correlation: College-educated individuals work more days per week
− 0.296	College, working hours/day	Work and education	Weak negative correlation: College-educated individuals work fewer hours per day

Most important features for work and career

- Workload: College students often balance academic work with part-time jobs, which can contribute to stress and pain.
- College-specific: Medical students (Code 3) work more days per week but fewer hours per day, which may contribute to stress and pain.

Table 15 summarizes key findings from the study, combining significant weight and health-related correlations with the strongest pain correlations specific to different fields of study. This integrated view helps highlight how both individual health indicators and academic disciplines influence weight patterns and pain experiences.

**Table 15:** Combined correlations of weight, health, and college-specific pain.

Correlation strength	Variables	Category	Description
+ 0.411	BMI, weight in kg	Weight and health	Moderate positive correlation: Higher BMI is associated with higher weight
+ 0.359	Sex, weight in kg	Weight and demographics	Moderate positive correlation: Males tend to have higher weight
+ 0.382	Scientific rank, weight in kg	Weight and career	Weak positive correlation: Higher scientific rank is associated with higher weight
− 0.268	Low back pain, college (physical therapy)	Pain and education (PT)	Moderate negative correlation: PT students report significantly less low back pain
− 0.210	Neck pain, college (physical therapy)	Pain and education (PT)	Moderate negative correlation: PT students report significantly less neck pain
+ 0.613	Wrist pain (last 7 days), wrist pain	Pain consistency (dentistry)	Strong positive correlation: Dentistry students report consistent wrist pain
+ 0.556	Wrist pain, wrist pain prevents activity	Pain impact (dentistry)	Moderate positive correlation: Wrist pain significantly limits activity for dentistry students
+ 0.588	Low back pain, low back pain (last 7 days)	Pain consistency (medicine)	Moderate positive correlation: Medical students report consistent low back pain
+ 0.508	Low back pain (7 days), low back pain prevents activity	Pain impact (medicine)	Moderate positive correlation: Recent low back pain significantly limits activity
+ 0.611	Hip pain, hip pain (last 7 days)	Pain consistency (pharmacy)	Moderate positive correlation: Pharmacy students report consistent hip pain
+ 0.598	Hip pain, hip pain prevents activity	Pain impact (pharmacy)	Moderate positive correlation: Hip pain significantly limits pharmacy students’ activities
+ 0.634	Thoracic pain (last 7 days), thoracic spine pain	Pain consistency (nursing)	Strong positive correlation: Nursing students report consistent thoracic pain
+ 0.503	Thoracic spine pain, thoracic pain prevents activity	Pain impact (nursing)	Moderate positive correlation: Thoracic pain significantly limits the ability to perform tasks

Summary of strong college-specific pain findings.

1. Physical therapy (code 1):
  Less low back and neck pain: PT students report significantly less low back and neck pain due to better posture, exercise habits, and ergonomic awareness.

2. Dentistry (code 2):
  Wrist Pain: Dentistry students report consistent wrist pain due to repetitive tasks, which significantly impacts their activity levels.

3. Medicine (code 3):
  Low Back Pain: Medical students report consistent low back pain due to long working hours, which significantly limits their activity.

4. Pharmacy (code 4):
  Hip pain: Pharmacy students report consistent hip pain due to prolonged standing, which significantly impacts their work and study.

5. Nursing (code 5):
  Thoracic pain: Nursing students report consistent thoracic pain due to physically demanding tasks, which significantly limits their ability to perform patient care.

Key insights

i. Physical therapy (code 1): Demonstrates the benefits of exercise and ergonomic training in reducing pain.
ii. Dentistry (code 2): Highlights the need for ergonomic interventions to address repetitive strain injuries.
iii. Medicine (code 3): Emphasizes the importance of managing workload and stress to reduce low back pain.
iv. Pharmacy (code 4): Suggests the need for breaks and proper footwear to mitigate hip pain from prolonged standing.
v. Nursing (code 5): Underscores the importance of proper lifting techniques and physical conditioning to reduce thoracic pain.

## Discussion and limitations

The present study introduced a new framework leveraging Particle Swarm Optimization (PSO) to enhance the classification and classification of musculoskeletal pain using machine learning. Our results demonstrated the effectiveness of integrating PSO with neural networks, achieving high classification accuracy across various pain types. This discussion interprets the key findings, compares them with existing literature, highlights practical implications, and acknowledges study limitations while suggesting future research directions.

### Key findings and interpretations

Our PSO-optimized neural network achieved remarkable performance, with accuracy ranging from 95.8 to 100% across different musculoskeletal pain types. Notably, elbow pain classification reached 100% accuracy, while hip pain (99.7%) and ankle pain (98.9%) also exhibited near-perfect classification. These results suggest that PSO effectively optimizes neural network weights and biases, improving the model’s ability to capture complex, non-linear relationships between predictors (e.g., age, BMI, exercise frequency, occupational factors) and pain outcomes.

The high F1 scores (95.4–100%) indicate a strong balance between precision and recall, minimizing false positives and false negatives. This is particularly crucial in clinical settings where misclassification could lead to inadequate interventions or unnecessary treatments. The AUC-ROC scores (95.5–100%) further confirm the model’s robustness in distinguishing between pain and non-pain cases.

### Clinical and practical implications

The identified pain determinants have significant implications for personalized interventions:

1. Exercise and pain reduction: Negative correlations between exercise frequency and knee/hip pain (− 0.188 and − 0.166, Table 8) suggest that targeted physical activity programs could mitigate pain in sedentary professionals.
2. Occupational factors: Prolonged work hours correlated with low back pain (Medicine, Code 3), while repetitive tasks linked to wrist pain (Dentistry, Code 2). Ergonomic adjustments and regular breaks could alleviate these issues.
3. BMI and pain: Higher BMI weakly correlated with knee pain (+ 0.142, Table 10), reinforcing the need for weight management in pain prevention strategies.

Our framework’s high accuracy enables early risk identification, allowing clinicians to design tailored rehabilitation programs (e.g., posture correction for neck pain in office workers) or policymakers to implement workplace health initiatives.

### Strengths and limitations

This study offers several key strengths that enhance the validity and applicability of its findings. First, the use of a comprehensive dataset comprising 350 participants from diverse professional backgrounds ensures robust generalizability across different occupational settings. Second, the integration of Particle Swarm Optimization (PSO) significantly improved neural network performance, surpassing traditional machine learning models in classification accuracy and reliability. Finally, the study adopted a holistic pain assessment framework, evaluating musculoskeletal pain across nine distinct body regions—a notable advancement over previous research that often focused on isolated areas. This multi-region approach provides a more complete understanding of occupation-related pain patterns.

Despite its strengths, this research has some limitations that should be acknowledged. First, the cross-sectional design restricts the ability to track pain progression over time; future studies could benefit from longitudinal data to assess temporal trends and causality. Second, pain data were self-reported, introducing potential recall bias. Incorporating wearable sensors or clinical assessments in future work could yield more objective measurements. Lastly, while the PSO-optimized model demonstrated superior accuracy, its computational cost (36–46 s per pain type) may hinder real-time deployment in clinical or industrial settings. Future optimizations could explore lightweight architectures or hybrid algorithms to balance performance and efficiency.

## Conclusions and future work

This study developed an advanced predictive framework for musculoskeletal pain assessment by integrating Particle Swarm Optimization (PSO) with neural networks, overcoming the limitations of conventional analytical approaches. By modeling the complex, non-linear interactions between occupational, demographic, physical, and lifestyle factors across a robust dataset of 350 participants, the framework achieved exceptional classification performance. The PSO-optimized neural network demonstrated remarkable accuracy (95.8–100%), with perfect discrimination for elbow pain (100%) and near-perfect results for hip pain (99.7%). Consistent precision (0.959–1.0), recall (0.956–1.0), and F1-scores (0.954–1.0) highlighted its balanced predictive capability, while AUC-ROC values (95.5–100%) confirmed superior class separability across all nine anatomical regions. These outcomes not only validate the model’s reliability in pain classification but also underscore its potential for clinical and occupational health applications. Future research could enhance generalizability through multicenter longitudinal data and reduce computational costs via hybrid optimization techniques. By addressing self-reporting biases with wearable sensor integration, subsequent iterations may further solidify the framework’s translational utility. This work establishes a foundation for AI-driven musculoskeletal pain management, bridging data science and preventive healthcare.

Future research should expand the dataset with diverse and longitudinal data, integrate wearable devices and electronic health records, and explore advanced machine learning techniques like deep learning and ensemble methods. Hybrid and adaptive optimization algorithms can enhance performance, while personalized interventions and real-time feedback systems improve pain management. Clinical validation and collaboration with healthcare providers are essential for practical application, and extending the framework to predict pain in additional body regions and incorporating severity metrics will provide a comprehensive understanding of musculoskeletal health. User-friendly interfaces, ethical considerations, and cost-effectiveness analyses will ensure responsible use, scalability, and economic impact, ultimately supporting improved musculoskeletal health outcomes and effective prevention strategies.

## Data Availability

The dataset and code used in this study are public and all test data are available at this portal (https://github.com/tarekhemdan/Musculoskeletal_Pain).

## Declarations

### Trial registration

This study employed a crosssectional design to examine workrelated musculoskeletal disorders among faculty members at universities in AlMinia Governorate, Egypt. Conducted between June and December 2024, the study protocol received ethical approval from Deraya University’s Institutional Review Board (Approval No. DCSR01002419). This investigation pursued two primary objectives assessing current prevalence rates of musculoskeletal disorders among academic staff and developing classification models for pain assessment. The methodology incorporated both populationlevel epidemiological analysis and individualized risk classification through standardized data collection procedures.
